# Short-Term Wearable Sensors for In-Hospital Medical and Surgical Patients: Mixed Methods Analysis of Patient Perspectives

**DOI:** 10.2196/18836

**Published:** 2021-04-22

**Authors:** Meera Joshi, Stephanie Archer, Abigail Morbi, Sonal Arora, Richard Kwasnicki, Hutan Ashrafian, Sadia Khan, Graham Cooke, Ara Darzi

**Affiliations:** 1 Department of Surgery and Cancer Imperial College London London United Kingdom; 2 Chelsea and Westminster Hospital NHS Foundation Trust London United Kingdom; 3 Department of Infectious Disease Wright-Fleming Institute London United Kingdom

**Keywords:** patient feedback, patient evaluation, questionnaire, interview, qualitative

## Abstract

**Background:**

Continuous vital sign monitoring using wearable sensors may enable early detection of patient deterioration and sepsis.

**Objective:**

This study aimed to explore patient experiences with wearable sensor technology and carry out continuous monitoring through questionnaire and interview studies in an acute hospital setting.

**Methods:**

Patients were recruited for a wearable sensor study and were asked to complete a 9-item questionnaire. Patients responses were evaluated using a Likert scale and with continuous variables. A subgroup of surgical patients wearing a Sensium Vital Sign Sensor was invited to participate in semistructured interviews. The Sensium wearable sensor measures the vital signs: heart rate, respiratory rate, and temperature. All interview data were subjected to thematic analysis.

**Results:**

Out of a total of 500 patients, 453 (90.6%) completed the questionnaire. Furthermore, 427 (85.4%) patients agreed that the wearable sensor was comfortable, 429 (85.8%) patients agreed to wear the patch again when in hospital, and 398 (79.6%) patients agreed to wear the patch at home. Overall, 12 surgical patients consented to the interviews. Five main themes of interest to patients emerged from the interviews: (1) centralized monitoring, (2) enhanced feelings of patient safety, (3) impact on nursing staff, (4) comfort and usability, and (5) future use and views on technology.

**Conclusions:**

Overall, the feedback from patients using wearable monitoring sensors was strongly positive with relatively few concerns raised. Patients felt that the wearable sensors would improve their sense of safety, relieve pressure on health care staff, and serve as a favorable aspect of future health care technology.

## Introduction

Delayed detection of patient deterioration in hospitals is a major cause of morbidity and mortality and is mostly caused by human-related monitoring failure [1-3]. Patients’ psychological parameters are altered during deterioration, particularly their vital signs, which are recognized early on [4,5]. Vital sign changes measured as part of routine clinical care for hospitalized patients may be present several hours prior to the onset of clinical events such as cardiac arrest, death, and intensive care unit admission [6]. Unfortunately, existing systems are unable to detect patient deterioration rapidly, and 39% of acute emergency patients admitted to critical care units are referred late [1].

In the United Kingdom, the National Early Warning Scoring (NEWS) System is used to detect clinical deterioration and improve patient safety. This is an aggregate scoring system that measures vital signs [3]. A score is allocated to each vital sign parameter, and high scores indicate patient deterioration [3]. If a patient has an NEWS of 0, a minimum of 12 hourly observations are recommended [3]. Among most patients in a general ward, observations are made 4-6 hours apart, but the frequency is increased for patients in a more critical condition [3]. Continuous invasive monitoring is only feasible in high-dependency units and not in the general ward setting where better noninvasive monitoring methods are needed. The latest lightweight sensors offer the potential for continuous monitoring of in-hospital patients.

Some previous studies have reviewed the reliability of wearable devices [4,5]. However more studies are needed to elucidate the performance of wearable devices [6] and to understand the perspectives of patients using them. The last few years have seen a drastic increase in wearable sensors in various clinical contexts from continuous monitoring during pregnancy [7] and assessment of patients with sleep apnea [8] and multiple sclerosis [9]. More studies have reviewed the patient adherence to and satisfaction with new technologies, with greater emphasis on patient feedback [9].

This study aimed to explore patient experiences with wearable sensor technology and carry out continuous monitoring through questionnaire and in-depth semistructured interviews in an acute hospital setting.

## Methods

### Study Design

A mixed methods approach was adopted to evaluate the breadth and depth of patient experience with a wearable sensor technology. To evaluate the breadth, patients recruited in a wearable patch study were asked to complete a questionnaire; semistructured interviews were held for a subgroup of patients to explore their experience with the sensor in detail.

### Ethics

Ethical approval was granted by Yorkshire & The Humber - Leeds East Research Ethics Committee (reference number 17/YH/0296).

### Patient Recruitment

All patients were recruited in a wearable patch study performed at West Middlesex University hospital—a busy hospital located in northwest London serving an ethnically diverse population. For recruited patients not understanding English (written or spoken), efforts were made to identify an appropriate translator to enable informed consent.

### Sensium Sensor

Acutely unwell patients admitted to hospital were provided the Sensium Vital Sign Sensor (The Surgical Company) in addition to undergoing standard monitoring of vital signs by nurses.

**Figure 1:**
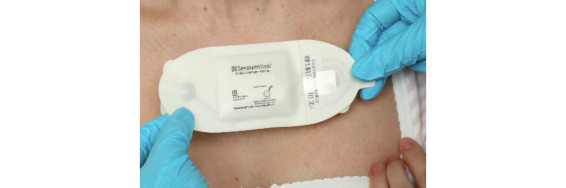
The Sensium wearable sensor being placed on a patient’s chest. The image was reproduced with permission from Sensium, Abingdon, UK.

The Sensium wearable sensor measures the vital signs: heart rate (HR), respiratory rate (RR), and temperature. The sensor is a one-off sensor with a battery life of 5 days; for longer hospitalization periods, an additional sensor is required. This sensor is lightweight, disposable, and waterproof. It transmits data wirelessly via low-power radio frequency signals to engineered bridges, which further transmit the data to a server. The data flow from the sensor to the virtual server via a bridge before being transmitted via Wi-Fi to smartphone apps. Figure 1 shows the sensor placement on a patient’s chest. The sensor was placed by either trained health care professionals looking after the patient or the research team. The patch was attached to the anterior chest wall, using two standard disposable electrocardiography (ECG) electrodes (Red-Dot2560, 3M Co). Surgical tape was used to secure the temperature probe in the axilla.

A previously reported predictive strategy is used to calculate the HR based on the RR interval [10]. The RR is derived from changes in thoracic impedance. A very small current is injected through the ECG electrodes. Changes in thoracic impedance are detected as variations in voltage (V) measured at the ECG electrodes. Inhalation (peak resistance) and exhalation (trough resistance) are detected from a 60-second segment of an IP waveform to calculate the median RR. Temperature is measured using a calibrated thermistor placed in the patient’s axilla. Individual vital sign parameters are measured and processed in a time-dependent manner.

**Figure 2:**
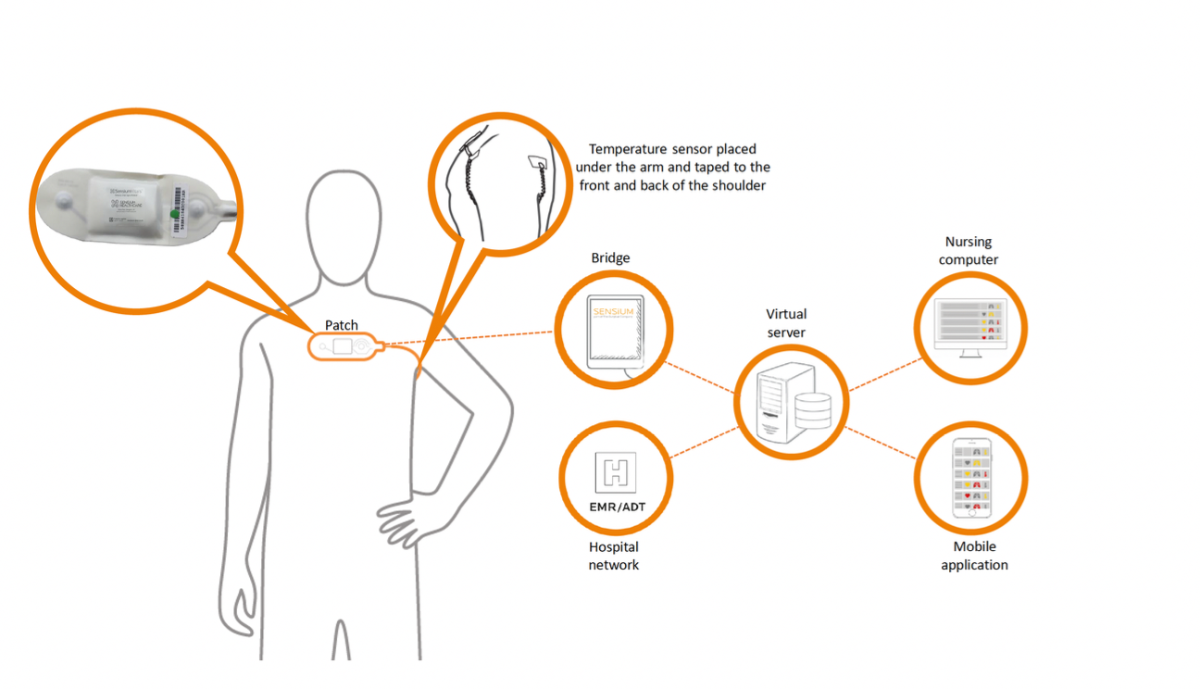
The Sensium wearable sensor demonstrating data transmission to the server and then to the mobile apps or computers. The image was reproduced with permission from Sensium, Abingdon, UK.

Once the vital signs are measured, it is transmitted to a microchip in the sensor, which has an inbuilt processing unit that transmits the average HR values as beats per minute and RR as breaths per minute, to the nearest bridge. These data are then transmitted to the central server [10], allowing digital alerts to be sent to health care staff through smartphones or electronic health records (Figure 2).

Data security is critical when using wearable technology. The Sensium system is ISO 27001 compliant, safe, and secure. The Sensium patches are uniquely identified through a machine-readable serial number, which can be matched to a patient ID band on the Sensium server with the use of a bar code scanner. No patient-identifiable information is communicated from the Sensium patch to the Sensium bridge, except for the device serial number and the HR, RR, and temperature values. After information transfer from the Sensium bridge to the secure Sensium server, the values derived from the patch are contextualized with patient-identifiable demographic information, which is usually obtained from the Patient Administration System. The Sensium patch transmits data to the Sensium bridge every 2 minutes and receives positive feedback from the Sensium server that the data have been received. If the Sensium bridge is out of range or the server yields no positive feedback, the patch continuously attempts communication until successful. The Sensium patch stores up to 3 hours of data locally and transmits this information to a Sensium bridge once back in range.

### Questionnaire

All patients who had worn the sensor during the wearable patch study were invited to complete a questionnaire, which was previously piloted with volunteers. The questionnaire was adapted from the Systems Usability Scale—a reliable tool for assessing the reliability of a device [11]. This 9-item questionnaire was scored on a 5-point Likert scale ranging from strongly agree, agree, neutral, disagree, and strongly disagree (1=strongly agree, 2=agree, 3=neutral, 4=disagree, 5=strongly disagree, and 6=no data). The questionnaire encompassed five main themes: patient comfort, understanding, safety, and whether patients would wear the device again in the hospital or at home. A copy of the questionnaire can be found in Multimedia Appendix 1. The questionnaire was a paper-based questionnaire completed after the hospitalization period on sensor removal.

### Semistructured Interviews

A subgroup of the recruited patients was invited to participate in an in-depth semistructured interview. All interviews were conducted by the lead researcher (MJ), using a prepared topic guide, which was previously evaluated by healthy volunteers. The questions were open-ended and focused on the following aspects: patient understanding, continuous monitoring, comfort, problems of any kind, potential future changes, and patient perception of potential future home monitoring technologies. An example of an interview question is “How do you feel about being monitored with the wearable sensor?” Interviews were audio-recorded and transcribed verbatim.

### Analysis

#### Questionnaire Data

Data were analyzed using SPSS (version 25, IBM Corp). Consistent with previous similar studies [12-14], the scores for each question were considered continuous variables. Means (SD) values were calculated per question.

#### Semistructured Interviews

Interview data were subjected to thematic analysis [15]. Thematic analysis facilitates the identification, analysis, and determination of reporting patterns (themes) within data sets and the organization and provision of depth. Initial codes were developed by MJ and were independently reviewed by a second coder (AM); these codes were discussed and refined until final themes were generated.

## Results

### Patient Demographics

In total, 453 of 500 (90.6%) patients who had worn the wearable sensor completed the questionnaire, of whom 231 (51%) were female, and the mean age was 57 (range 18-95) years. The sample was representative of the overall study population. All patients wore the sensor throughout their stay of 2 days on average.

A total of 12 patients (male: n=6, 50%) participated in the semistructured interviews. Patients were recruited from various medical and surgical admitting wards, and their mean age was 49 (range 23-73) years. Detailed patient information is provided in Table 1.

**Table 1:** Patient demographics.

Patient #	Specialty	Sex	Age (years)	Employment status	Ethnicity	Presenting complaint	Frailty	Residence	Past medical history	Infection
1	Surgical	M	71	Retired	White, British	Deranged renal function, high stoma output	Independent	Own home	Laparotomy, gall bladder and ascending colon removed 11/2017, post op leak and further laparotomy 3 weeks later	N
2	Surgical	M	25	Student	White, British	Perforation, had a laparotomy	Independent	Own home	N/A^a^	Y
3	Surgical	M	20	Employed	White, British	Appendicitis	Independent	Own home	Cyst removed from Jaw	Y
4	Surgical	F	33	Self-employed	Other White background	Acute cholecystitis	independent	Own home	N/A	Y
5	Surgical	F	27	Employed	Mixed background	Terminal ileal Crohn disease	Independent	Own home	N/A	Y
6	Surgical	F	48	Employed	White, British	Appendicitis, had a laparotomy, thought to be Meckel’s	Independent	Own home	N/A	Y
7	Surgical	F	73	Retired	White, British	ERCP^b^ pancreatitis	Independent walks with crutches (amputee has prosthesis)	Own home	N/A	N
8	Surgical	M	66	Employed	White, British	Cholecystitis/biliary colic	Independent	Own home	N/A	N
9	Surgical	M	70	Employed	Other White background	Irreducible paraumbilical hernia, abdominal pain	Independent	own home	N/A	Y
10	Surgical	F	54	Employed	White, British	Unwell and diarrhea	Independent	Own home	Appendicectomy, hysterectomy, breast lump removal	Y
11	Surgical	M	74	Unknown	White, British	Rib fracture falling off a ladder	Independent	Own home	N/A	N
12	Surgical	F	40	Employed	Black/Black, British African	Subacute bowel obstruction	Independent	Own home	Several bowel operations, repeated bowel obstructions	N

^a^N/A: not applicable.

^b^ERCP: endoscopic retrograde cholangiopancreatography.

### Questionnaire Outcomes

The questionnaire results were positive overall. Descriptive statistics for each questionnaire item are provided in Table 2. In total, 427 of 500 (85.4%) patients agreed that the wearable sensor was comfortable to wear and 27 (5.4%) reported that the sensor was cumbersome. Only 11 (2.2%) patients thought the system was complex, while 416 (83.2%) agreed that they knew whom to contact if problems arose. Furthermore, 397 (79.4%) patients did not feel the need for extensive information before sensor use. The majority of patients (n=445, 89.0%) agreed that they understood the purpose of the wearable sensor and 347 (69.2%) felt safer being monitored. Regarding future use, most patients agreed that they would wear the sensor again when in the hospital (n=429, 85.8%) and at home (n=398, 79.6%).

**Table 2:** Descriptive statistics for each questionnaire item.

Please rate your level of agreement with the following statements	Strongly agree, n (%)	Agree, n (%)	Neutral, n (%)	Disagree, n (%)	Strongly disagree, n (%)	No data, n (%)	Total number, n (%)
1. The wearable patch was comfortable to wear	224 (44.8)	203 (40.6)	14 (2.8)	11 (2.2)	1 (0.2)	47 (9.4)	500 (100)
2. I found this system unnecessarily complex	1 (0.2)	10 (2.0)	12 (2.4)	277 (55.4)	152 (30.4)	48 (9.6)	500 (100)
3. I understood what the wearable patch was for	213 (42.6)	232 (46.4)	4 (0.8)	3 (0.6)	0 (0.0)	48 (9.6)	500 (100)
4. I felt safer being monitored whilst wearing the wearable patch	132 (26.4)	214 (42.8)	90 (18.0)	16 (3.2)	0 (0.0)	48 (9.6)	500 (100)
5. I knew who to contact if I had any problems with the wearable patch	154 (30.8)	262 (52.4)	15 (3.0)	19 (3.8)	2 (0.4)	48 (9.6)	500 (100)
6. I would wear the wearable patch again when in hospital	175 (35.0)	254 (50.8)	12 (2.4)	9 (1.8)	0 (0.0)	50 (10.0)	500 (100)
7. I found this system very cumbersome to wear	4 (0.85)	23 (4.6)	19 (3.8)	263 (52.6)	142 (28.4)	49 (9.8)	500 (100)
8. I would wear the wearable patch in my home	163 (32.6)	235 (47.0)	17 (3.4)	30 (6.0)	6 (1.2)	49 (9.8)	500 (100)
9. I needed to learn a lot of things before I could get going with this system	11 (2.2)	32 (6.4)	11 (2.2)	263 (52.6)	134 (26.8)	49 (9.8)	500 (100)

### Semistructured Interview Outcomes

**Figure 3:**
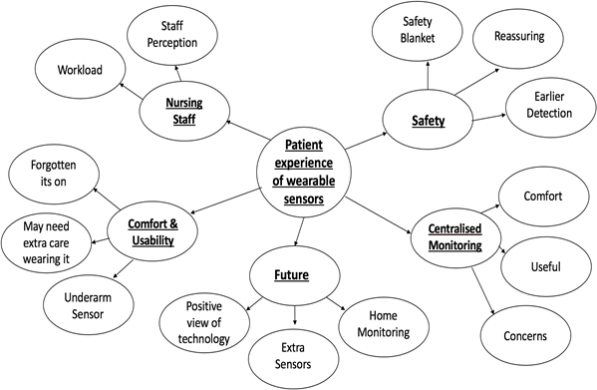
Themes and subthemes from thematic analysis.

Five main themes emerged from the interviews: (1) centralized monitoring, (2) safety, (3) impact on nursing staff, (4) comfort and usability, and (5) the future and views on technology. These themes and their contributing subthemes are summarized in Figure 3.

### Centralized Monitoring

Centralized monitoring was consistently described by patients and included many of the other themes. Centralized monitoring provided patients with greater peace of mind, and patients felt that health care staff were always around if a problem arose.

Remote monitoring so your doctor can sit at the desk and see his patients, how you’re doing.

Patient 9, male, aged 70 years

Patients described feeling reassured knowing that they were being monitored even when health care staff were not at their bedside.

I think it would be really reassuring for people, especially as the data is centralized.

Patient 10, female, aged 54 years

However, one patient was concerned about the patch potentially not allowing for any monitoring as no feedback was provided to the patients.

I was worried it wasn’t really recording when it needed to be recording.

Patient 8, male, aged 66 years

Central monitoring was discussed among all interviewed patients, which provided patients with greater peace of mind and reassurance. One patient was concerned about the lack of feedback to patients.

### Safety

All interviewed patients commented on an enhanced feeling of patient safety while wearing the patch. The extra layer of support provided them a feeling of reassurance.

I felt like there was a second safety blanket around me, almost, and that I was constantly in amongst the nurses. I appreciate that the nurses do their obs as frequently as they can, but they’re very busy. So, for this to be on constantly, it’s reassuring.

Patient 2, male, aged 25 years

Problems with the current monitoring system were identified from their experiences in the ward. This was particularly noticeable overnight, where the perceived current monitoring by nurses was minimized.

Reassuring, an extra layer, particularly at night where they don’t do obs.

Patient 10, female, aged 54 years

In some patients requiring indwelling lines (ie, nasogastric tube), a patient’s speech ability seemed to prevent them from being able to raise concerns if there was a problem. Patients felt that the additional use of the wearable patch along with continuous monitoring could help provide patients with an additional “voice” if their condition suddenly exacerbated.

Some people can’t talk like if they’ve got tubes in and stuff but that could talk for them, like if they can’t say I’m feeling hot or feeling ill.

Patient 12, female, aged 40 years

Patient-identified high-risk groups that may benefit the most from the wearable sensors included the following: children, postoperative patients, and the elderly. Patients were concerned that current systems may cause a delay in identifying unwell patients, thus compromising patient safety. Conversely, patients believed that continuous monitoring for conditions such as infection would improve patient safety and facilitate earlier detection of patient deterioration.

### Nursing Staff

The impact of continuous monitoring on staff workload was highlighted. Most patients believed that the technology would reduce the workload of nurses who were already perceived to be under considerable strain.

I missed my medication dose because they were so busy, and they didn’t get to me. I know it’s not about medicine but they would have been alerted on their, you know, say if my temperature had gone up or – It’s just the fact that they’re so busy and there’s not enough of them, I think it’s good, it can take some of the strain off of their workload.

Patient 12, female, aged 40 years

The patients understood the demanding nature of a nurse’s role and that staff shortages add further pressure on an already stretched workforce. Patients felt that wearable sensors would improve the nursing workload and allow them time to take on other tasks.

Free nurses for an awful lot of routine stuff, you know they have been doing me every half an hour, am sure there are better things they can be doing in a busy place like this.

Patient 9, male, aged 70 years

While most patients agreed that the wearable sensors would ease the nursing workload, one patient was concerned that continuous monitoring may increase the demands placed on staff.

The nurses might be running away if they have got too much to do.

Patient 8, male, aged 53 years

Another concern of one patient was that if the sensors were too efficient, they would be used as a replacement for a regular check-up by a nurse.

Thought it was to see if it could replace the need to have regular nurse check-ups of heart rate and what not.

Patient 3, male, aged 20 years

In summary, all patients reported that wearable sensors and continuous monitoring would affect the nursing workload. Most patients believed that these would ease the nursing workload; however, one patient was concerned that the demands on the staff would be increased, and another patient was concerned that sensors may be used as a replacement for regular check-ups by nurses if they prove to be too efficient.

### Comfort and Usability

All patients described the patch as being comfortable to wear. Many patients had forgotten about the existence of the patch on their person once it was on.

From about five minutes after it was on, I completely forgot it was there.

Patient 2, male, aged 25 years

One patient described the sensor as being very comfortable, but slightly irritating after a week.

I just find it a bit irritating now when I have to adjust it every now and then, but I had it on for a week.

Patient 8, male, aged 66 years

Certain everyday tasks, such as changing clothes and bathing required extra care to prevent dislodging the patch and reducing monitoring.

Only concern was when I wanted to get changed, I didn’t want to sort of move it off too quickly, in case I caught on the sensor when I had a wash.

Patient 6, female, aged 48 years

During interviews, patients made some suggestions to enhance the comfort levels of the patch and to improve overall usability. A few patients indicated that the patch could be made smaller but were concerned that doing so would potentially reduce comfort levels. Other potential changes identified for both utility and comfort were changes to the fixings and the underarm temperature sensor.

The underarm sensor I managed to knock off in the shower so that might need some looking at, caught the spiral cable when towel drying, I knocked the patch off, fixings comparatively bulky.

Patient 1, male, aged 71 years

Overall, all patients agreed that the sensor was comfortable. Those patients who had worn the sensor for longer a period—up to 1 week—found it to be irritating at times. Certain everyday tasks such as bathing and changing clothes required additional care with the sensor on. Patients made several suggestions to improve the sensor further, which included a size reduction and changes to the underarm temperature sensor.

### The Future

Patients described wearable sensors as the future and being a “step forward” [Patient 3, male, aged 20 years]. In future, all patients are likely to wear the sensor while at home. High-risk groups such as children and postoperative patients were identified to most benefit from home-based monitoring, and they agreed that this constituted an enhancement in medical care technology. Patient 1 (male, aged 71 years) stated, “I think it would be brilliant if we can extend early developments into the home environment,” further stating that these developments would help in “flagging dangerous symptoms.”

The use of sensors for home-based monitoring was particularly important because patients expressed their current concern of going home after an operation and becoming unwell. Moreover, one patient described how her friend developed an infection at home post surgery and died.

She developed an infection post her cancer surgery. But, so it wasn’t even the actual surgery that killed her as such, but you know like these symptoms come on really quickly, you’re starting to feel – because you get delirious when you get a temperature and you may not realise how sick you are, but if that was to send a message to somebody then I just think that’s amazing.

Patient 12, female, aged 40 years

Patients felt that future sensors would be smaller and have additional features such as blood pressure monitoring and accelerometry, which would help detect falls, particularly for high-risk elderly individuals at home.

Yes, I would imagine for old people it could be very valuable, particularly if it detected movement as well.

Patient 9, male, aged 70 years

Patients suggested further sensor modalities such as implantable sensors for future use. They felt that this would be the natural evolution of sensor technology and would be beneficial.

Overall, patients expressed a positive opinion of technology in general and were reassured with the technological advancement in health care.

## Discussion

### Study Overview

A key determinant to the further use of wearable devices is end-user evaluation by patients. To date, limited qualitative data on patient evaluation are available within the wider literature, particularly from among patients in acute hospital settings. Researchers have reported that larger sample sizes are required for evaluating future wearable sensors [16]. To our knowledge, this is the largest questionnaire study of wearable sensors used in an acute hospital setting to date. The large questionnaire sample size coupled with in-depth patient interviews helped ensure diversity in the responses, providing future insights into patient perspectives.

### Principal Findings

#### Safety

This study reported that most patients felt safe wearing the patch, describing it as an “extra safety blanket.” Patients felt safer with centralized monitoring systems, concurrent with a previous study describing how sensors provide patients with a sense of added security [17].

#### Comfort

Most patients in this study found the wearable sensor comfortable, and many of them reported they had forgotten that they were wearing it. Similar findings were reported in a previous study on elective surgical patients using the Sensium sensor where the sensor was so comfortable that the patients had forgotten that it was on (10 of 12 patients) [18]. Patient comfort with wearable sensors was assessed through interviews, which has also been reported previously [18]. While this study reviewed the opinions of patients within an elective setting the opinion of patients in an acute setting have thus far remained unexplored. A questionnaire study on the use of wearable devices at home to assess seizures in patients reported that patient comfort levels are an important consideration [19].

#### Ease of Sensor Use and Design

Sensor design and simplicity of use are important. Most patients in this study did not require extensive information before using the technology, reflecting the ease of use among patients. Previous studies have revealed that technical problems and complicated designs can cause frustration and stress among patients [20]. A simple sensor design is an important end-user preference [8].

#### Potential for Further Use of Wearable Sensors Both in the Hospital and at Home

In this study, most patients would wear the wearable sensors again when in the hospital and at home. High demands among patients would potentially assist the future use of wearable sensors. Patients had a very positive view of the sensor technology overall and felt that wearable sensors facilitating continuous monitoring would certainly be used in the future. The concept of home-based monitoring using wearable sensors was welcomed by patients and is likely the next step in wearable sensing technology. A small study using a wearable sensor called Vital Connect in the home setting has been previously reported [20]. This study reports “encouraging positive feedback on wearability and usability” of the sensors for home use [20]. High-risk groups potentially benefiting the most were identified by patients in this study, including children, the elderly, and postoperative patients. Wearable sensor use by the elderly is gaining increasing interest [21]. Studies on the use of vital sign monitors coupled with additional monitoring sensors such as fall detectors and physical activity monitors are currently underway [22]. In cases of deterioration, alerts would be sent to family members or caregivers [10]. With a worldwide ageing population, wearable sensor technologies may help generate solutions to provide support to individuals at home to facilitate independent living.

#### Concerns for Wearable Sensor Use

On interviewing patients, few concerns were raised about wearable sensors, but these concerns were raised by only a few patients. One patient reported that while she welcomed the monitoring tool, she did not want it to be an excuse to discharge patients early from hospital. Another patient expressed concerns over data security, which has also been previously highlighted [18].

#### Ideas for Future Wearable Sensor Development

Patients reported numerous areas for future development. These included a reduction in sensor size and changes to the design of the underarm sensor. This is concurrent with the wider literature; a review of patient perspectives of wearable sensors reported that patients preferred small, compact devices that were not directly visible to others to reduce any stigmatization [6].

### Strengths

This study lays the foundation for patients’ perspectives on wearable sensors in an acute hospital setting. Until now, a limited number of studies have reviewed patient feedback on wearable sensors. Unlike this study, previous studies have reviewed patient feedback as a secondary, rather than primary, objective [6]. A key strength of this study is the high rates of questionnaire completion with feedback from 453 of 500 (90.6%) patients. This enhances the reliability of the feedback generated and helps reduce any potential bias. The themes and subthemes derived from the interviews remarkably overlapped with the outcomes of the questionnaire, encouraging the use of both methods. Multiple data collection methods offered a comprehensive view of patient feedback.

### Limitations

There is a potential bias in patient recruitment as we can only obtain feedback from patients agreeing to wear the sensor. As such, these patients may have a positive opinion of technology compared to others not recruited in this study (a total of 1398 patients were screened, 691 were ineligible for recruitment, and 207 did not consent to participate in the trial). The average number of days for sensor use in this study was 2 days. Those wearing the sensor for prolonged periods may yield different outcomes particularly regarding comfort and usability. Only one sensor device (Sensium wearable sensor) was used in the study. Though the broad themes of sensor technology and continuous monitoring also apply to other sensors, themes such as comfort may not be applicable to other sensor devices.

### Future Perspectives

Further studies may reveal the opinions of patient’s friends and relatives to understand their perspectives on the technology. This would be of great importance if the wearable sensors were being used at home and alerting the patients’ family members. Future studies reviewing wearable sensor use over longer periods are required.

### Conclusion

Overall, the feedback from patients was strongly positive. Wearable sensor technology continues to develop, and these data suggest that patients would welcome its use when acutely unwell and in an acute hospital setting.

## Appendix

Multimedia Appendix 1 Patient questionnaire.

## Abbreviations

ECG: electrocardiography
HR: heart rate
IP: impedance pneumography
NEWS: National Early Warning Scoring
RR: respiratory rate
