# Supplementary material for: Short-Term Wearable Sensors for In-Hospital Medical and Surgical Patients: Mixed Methods Analysis of Patient Perspectives
Source: JMIR Perioper Med. 2021 Apr 22;4(1):e18836. doi: 10.2196/18836 (PMC8103292; doi:10.2196/18836)
Supplement: Multimedia Appendix 1 [file periop_v4i1e18836_app1.docx]

**WEARABLE PATCH VITAL SIGN MONITORING PROJECT**

**Participant Questionnaire on Wearable Vitals Patch Monitoring**

|  |  | Yes No |
| --- | --- | --- |
| **I received an information sheet prior to applying the wearable patch and was invited to ask questions?** |  |  |

**Please tick the appropriate boxes below on a scale from strongly agree to strongly disagree.**

| Please rate your level of agreement with the following statements | Strongly Agree | Agree | Neutral | Disagree | Strongly Disagree |  |
| --- | --- | --- | --- | --- | --- | --- |
| 1. The wearable patch was comfortable to wear |  |  |  |  |  |  |
| 1. I found this system unnecessarily complex |  |  |  |  |  |  |
| 1. I understood what the wearable patch was for |  |  |  |  |  |  |
| 1. I felt safer being monitored whilst wearing the wearable patch |  |  |  |  |  |  |
| 1. I knew who to contact if I had any problems with the wearable patch |  |  |  |  |  |  |
| 1. I would wear the wearable patch again when in hospital |  |  |  |  |  |  |
| 1. I found this system very cumbersome to wear |  |  |  |  |  |  |
| 1. I would wear the wearable patch in my home |  |  |  |  |  |  |
| 1. I needed to learn a lot of things before I could get going with this system |  |  |  |  |  |  |
| We welcome any additional comments you may have:    THANK YOU | | | | | | |

**Contact**

**Dr Meera Joshi Clinical Research Fellow & Surgical Registrar**, 10^th^ Floor QEQM Building, St Mary’s Hospital, Praed Street, London, W2 1NY, United Kingdom, Email [meera.joshi03@imperial.ac.uk](mailto:meera.joshi03@imperial.ac.uk)

**Patient Advice and Liaison Service (PALS)** West Middlesex University Hospital, Twickenham Road, Isleworth, TN7 6AF, Telephone 020 8321 6261

**Patient Advice and Liaison Service (PALS)** Ground floor of the Queen Elizabeth the Queen Mother (QEQM) building, St Mary’s Hospital, South Wharf Road, London W2 1NY. Telephone 020 3312 7777
